# Supplementary material for: Comprehensive Evaluation and Construction of Drought Resistance Index System in Hulless Barley Seedlings
Source: Int J Mol Sci. 2025 Apr 17;26(8):3799. doi: 10.3390/ijms26083799 (PMC12027527; doi:10.3390/ijms26083799)
Supplement: Supplementary file 1 [file ijms-26-03799-s001.zip › ijms-3525686-supplementary.pdf]

Supplementary table S1. Names and sources of hulless barley varieties

Number	Variety name	Variety origin	Drought-resistant ability
YC85	Zangqing 320	Lhasa Tibet	no
YC88	Zangqing 2000	Lhasa Tibet	no
ZY1252	Ziqingke	Lhasa Tibet	yes
ZY1100	Guoluo	Milin Nyingchi	yes
ZY97	Duobujiu	Rikaze Tibet	yes
YC83	Zangqing 148	Lhasa Tibet	no
ZY673	Naina	Linzhou Lhasa	unknown
ZY1403	Lanqingke	Jiangda Chamdo	unknown
KL14	Kunlun14	Qinghai	unknown
